# Supplementary material for: Development and comparison of novel multiple cross displacement amplification (MCDA) assays with other nucleic acid amplification methods for SARS-CoV-2 detection
Source: Sci Rep. 2021 Jan 21;11:1873. doi: 10.1038/s41598-021-81518-8 (PMC7819982; doi:10.1038/s41598-021-81518-8)
Supplement: Supplementary file 1 — Supplementary Information 1. [file 41598_2021_81518_MOESM1_ESM.pdf]

Development and comparison of novel multiple cross displacement amplification (MCDA) assays with other nucleic acid amplification methods for SARS-CoV-2 detection

Laurence Don Wai Luu, Michael Payne, Xiaomei Zhang, Lijuan Luo and Ruiting Lan

Supplementary Table 1: List of MCDA, LAMP and rt-PCR primers used in this study. Bolded MCDA primer names are primers shared between 2 or more primer sets. * indicate primers used in rt-PCR for confirmation of complete DNA removal from transcribed RNA. Red text indicates the final primer sets chosen for the MCDA SARS-CoV-2 assay.

MCDA Primer name	Sequence 5' --> 3'	Reference
**Region 1 ORF1ab primer set 1**		
R1_1_F1*	CTCATGTGGGCGAAATACC	This study
**R1_1_2_F2**	ATCGACATAGCGAGTGTATG	
**R1_1_2_C1**	TTTAGATCGGCGCCGTAACTATGG	
**R1_1_2_C2**	AAGAAAACTGGAACACTAAACATAGC	
**R1_1_2_D1**	CACCAGCTCCTTTATTACCG	
R1_1_D2	AGTGGTGTTACCCGTGAACT	
**R1_1_4_R1***	TCGTCGCCTAAGTCAAATGAC	
R1_1_2_R2	TTGGCACTGATCCTTATGAAG	
R1_1_2_CP1	TTTAGATCGGCGCCGTAACTATGGGCAAGGTTCTTCTTCGTAAGA	
R1_1_CP2	AGAAAACTGGAACACTAAACATAGCCTCCGTTAAGCTCACGCAT	
**Region 1 ORF1ab primer set 2**		
R1_2_F1	ATGTGGGCGAAATACCAGTG	This study
**R1_1_2_F2**	ATCGACATAGCGAGTGTATG	
**R1_1_2_C1**	TTTAGATCGGCGCCGTAACTATGG	
**R1_1_2_C2**	AAGAAAACTGGAACACTAAACATAGC	
**R1_1_2_D1**	CACCAGCTCCTTTATTACCG	
R1_2_D2	GTGGTGTTACCCGTGAACTC	
R1_2_R1	CGTCGCCTAAGTCAAATGAC	
**R1_1_2_R2**	TTGGCACTGATCCTTATGAAG	
**R1_1_2_CP1**	TTTAGATCGGCGCCGTAACTATGGGCAAGGTTCTTCTTCGTAAGA	
R1_2_CP2	AGAAAACTGGAACACTAAACATAGCCCCCTCCGTTAAGCTCACG	
**Region 1 ORF1ab primer set 3**		
R1_3_F1	TTATGGTTGAGCTGGTAGC	This study
**R1_3_4_F2**	TGTTATCGACATAGCGAGTGTA	
R1_3_C1	GCTCCTTTATTACCGTTCTTACGAAG	
R1_3_C2	TGGCACTGATCCTTATGAAGATTTTC	
R1_3_D1	ACTGGTATTTCGCCCACATGA	
R1_3_D2	CTGGAACACTAAACATAGCAGTG	
R1_3_R1	ATCGGCGCCGTAACTATGGCCA	
R1_3_R2	TCATTTGACTTAGGCGACGA	
R1_3_CP1	CTCCTTTATTACCGTTCTTACGAAGAGAACTCGAAGGCATTCAGT	
R1_3_CP2	GGCACTGATCCTTATGAAGATTTTCCCTCCGTTAAGCTCACGCATC	
**Region 1 ORF1ab primer set 4**		
R1_4_F1	AGAACTCGAAGGCATTCAGTA	This study
**R1_3_4_F2**	TGTTATCGACATAGCGAGTGTA	
R1_4_C1	TTAGATCGGCGCCGTAACTATGG	
R1_4_C2	CAAGAAAACTGGAACACTAAACATAG	
R1_4_D1	CTTACGAAGAAGAACCTTGC	
R1_4_D2	CAGTGGTGTTACCCGTGAACT	
**R1_1_4_R1**	TCGTCGCCTAAGTCAAATGAC	
R1_4_R2	TTGGCACTGATCCTTATGAAGAT	
R1_4_CP1	TTAGATCGGCGCCGTAACTATGGCTCATGTGGGCGAAATACCAGT	
R1_4_CP2	AAGAAAACTGGAACACTAAACATAGCCTCCGTTAAGCTCACGCAT	
**Region 2 ORF1ab primer set 1**		
**R2_1_2_F1***	CCACAGTACGTCTACAAGC	This study
**R2_1_2_F2**	TATGTGGCAACGGCAGTAC	
R2_1_C1	GTAAGCTTTAGCAGCATCTACAGC	
**R2_1_3_4_C2**	TGGTCAGGCAATAACAGTTACACC	
**R2_1_2_D1**	GCACAGAAAGATAATACAGTTG	
**R2_1_2_D2**	AAGCCAATATGGATCAAGAATC	
R2_1_R1*	AGTGATTGGTTGTCCCCCAC	
R2_1_R2	AGATGTTGTGTACACACACTG	
R2_1_CP1	GTAAGCTTTAGCAGCATCTACAGCTAATGCAACAGAAGTGCCTGC	
R2_1_CP2	TGGTCAGGCAATAACAGTTACACCAGACAACACGATGCACCACC	

Region 2 ORF1ab primer set 2

**R2_1_2_F1**	CCACAGTACGTCTACAAGC	This study
**R2_1_2_F2**	TATGTGGCAACGGCAGTAC	
R2_2_C1	CTTTGTAAGCTTTAGCAGCATCTAC	
R2_2_C2	GGTCAGGCAATAACAGTTACACCG	
**R2_1_2_D1**	GCACAGAAAGATAATACAGTTG	
**R2_1_2_D2**	AAGCCAATATGGATCAAGAATC	
R2_2_R1	CAATTAGTGATTGGTTGTCCC	
R2_2_R2	GATGTTGTGTACACACACTGG	
R2_2_CP1	TTTGTAAGCTTTAGCAGCATCTACGTAATGCAACAGAAGTGCCT	
R2_2_CP2	GGTCAGGCAATAACAGTTACACCGAGACAACACGATGCACCAC	

Region 2 ORF1ab primer set 3

R2_3_F1	AGTTTAGCTGCCACAGTACGTC	This study
**R2_3_4_F2**	CTATGTGGCAACGGCAGTACA	
R2_3_C1	GTAAGCTTTAGCAGCATCTACAGCAA	
**R2_1_3_4_C2**	TGGTCAGGCAATAACAGTTACACC	
R2_3_D1	GCACAGAAAGATAATACAGTTGAAT	
**R2_3_4_D2**	GGAAGCCAATATGGATCAAGAA	
**R2_3_4_R1**	ACACAATTAGTGATTGGTTGTCC	
R2_3_R2	AGATGTTGTGTACACACACTGGT	
R2_3_CP1	AGCTTTAGCAGCATCTACAGCAAGGTAATGCAACAGAAGTGCC	
**R2_3_4_CP2**	GGTCAGGCAATAACAGTTACACCGACAACACGATGCACCACC	

Region 2 ORF1ab primer set 4

R2_4_F1	CCTAAATAGAGGTATGGTACTT	This study
**R2_3_4_F2**	CTATGTGGCAACGGCAGTACA	
R2_4_C1	TGTAAGCTTTAGCAGCATCTACAGCA	
**R2_1_3_4_C2**	TGGTCAGGCAATAACAGTTACACC	
R2_4_D1	GCAGGCACTTCTGTTGCATTACC	
**R2_3_4_D2**	GGAAGCCAATATGGATCAAGAA	
**R2_3_4_R1**	ACACAATTAGTGATTGGTTGTCC	
R2_4_R2	TAAGATGTTGTGTACACACACTG	
R2_4_CP1	TAAGCTTTAGCAGCATCTACAGCAAGTTTAGCTGCCACAGTACG	
**R2_3_4_CP2**	GGTCAGGCAATAACAGTTACACCGACAACACGATGCACCACC	

Region 3 N gene primer set 1

R3_1_F1*	AGAATGGAGAACGCAGTGG	This study
R3_1_F2	AAATACCATCTTGGACTGAG	
R3_1_C1	TGCCATGTTGAGTGAGAGCGGTG	
R3_1_C2	TAGCAGTCCAGATGACCAAATTGG	
R3_1_D1	AACCAAGACGCAGTATTATTG	
R3_1_D2	CTACCGAAGAGCTACCAGAC	
R3_1_R1*	CCTCGAGGGAATTTAAGGTC	
R3_1_R2	AAGGCGTTCCAATTAACACC	
R3_1_CP1	TGCCATGTTGAGTGAGAGCGGTGAAACAACGTCGGCCCCAAGG	
R3_1_CP2	AGCAGTCCAGATGACCAAATTGGTACCGTCACCACCACGAATT	

Region 3 N gene primer set 2

R3_2_F1	AAACAACGTCGGCCCCAAG	This study
R3_2_F2	TGGCCCAGTTCCTAGGTAG	
R3_2_C1	TTGTCCTCGAGGGAATTTAAGGTC	
**R3_2_3_C2**	ACCGAAGAGCTACCAGACGAATTC	
R3_2_D1	TTGCCATGTTGAGTGAGAGC	
R3_2_D2	GTGGTGGTGACGGTAAAATG	
R3_2_R1	CTATTGGTGTTAATTGGAACG	
R3_2_R2	CAGATGACCAAATTGGCTAC	
R3_2_CP1	TTGTCCTCGAGGGAATTTAAGGTCAATACTGCGTCTTGGTTCAC	
R3_2_CP2	CCGAAGAGCTACCAGACGAATTCAGAAATACCATCTTGGACTGA	

Region 3 N gene primer set 3

R3_3_F1	CGATCAAAACAACGTCGGCC	This study
R3_3_F2	TCTGGCCCAGTTCCTAGGTA	
R3_3_C1	TTGTCCTCGAGGGAATTTAAGGTCTT	
**R3_2_3_C2**	ACCGAAGAGCTACCAGACGAATTC	
R3_3_D1	CTTGCCATGTTGAGTGAGAGC	
R3_3_D2	GTGGTGGTGACGGTAAAATGAA	
R3_3_R1	CTATTGGTGTTAATTGGAACGCC	
R3_3_R2	TCCAGATGACCAAATTGGCTAC	
R3_3_CP1	TCCTCGAGGGAATTTAAGGTCTTCAATAATACTGCGTCTTGGTT	
R3_3_CP2	CGAAGAGCTACCAGACGAATTCTAGAAATACCATCTTGGACTGA	

Region 3 N gene primer set 4		
R3_4_F1	GCAGTAACCAGAATGGAGAAC	This study
R3_4_F2	TTCTGGCCCAGTTCCTAGGTA	
R3_4_C1	CTTGCCATGTTGAGTGAGAGCGGT	
R3_4_C2	CAAATTGGCTACTACCGAAGAGCTAC	
R3_4_D1	GAACCAAGACGCAGTATTATTG	
R3_4_D2	AATTCGTGGTGGTGACGGTAA	
R3_4_R1	TCCTCGAGGGAATTTAAGGTCT	
R3_4_R2	AAGGCGTTCCAATTAACACCAATA	
R3_4_CP1	[illegible]TTGCCATGTTGAGTGAGAGCGGTCAAAACAACGTCGGCCCCAA[illegible]	
R3_4_CP2	[illegible]ATTGGCTACTACCGAAGAGCTACTAGAAATACCATCTTGGACTG[illegible]	

LAMP primer name	Sequence 5' --> 3'	
**Region 1 ORF1ab primer set**		
ORF1a-A-F3	CTGCACCTCATGGTCATGTT	Zhang et al. 2020 Rapid Molecular Detection of SARS-CoV-2 (COVID-19) Virus RNA Using Colorimetric LAMP
ORF1a-A-B3	AGCTCGTCGCCTAAGTCAA	
ORF1a-A-FIP	GAGGGACAAGGACACCAAGTGTATGGTTGAGCTGGTAGCAGA	
ORF1a-A-BIP	CCAGTGGCTTACCGCAAGGTTTTAGATCGGCGCCGTAAC	
ORF1a-A-LF	CCGTACTGAATGCCTTCGAGT	
ORF1a-A-LB	TTCGTAAGAACGGTAATAAAGGAGC	

Region 3 N gene primer set		
GeneN-A-F3	TGGCTACTACCGAAGAGCT	Zhang et al. 2020 Rapid Molecular Detection of SARS-CoV-2 (COVID-19) Virus RNA Using Colorimetric LAMP
GeneN-A-B3	TGCAGCATTGTTAGCAGGAT	
GeneN-A-FIP	TCTGGCCCAGTTCCTAGGTAGTCCAGACGAATTCGTGGTGG	
GeneN-A-BIP	AGACGGCATCATATGGGTTGCACGGGTGCCAATGTGATCT	
GeneN-A-LF	GGACTGAGATCTTTCATTTTACCGT	
GeneN-A-LB	ACTGAGGGAGCCTTGAATACA	

rt-PCR primer name	Sequence 5' --> 3'	
**Region 3 N gene primer set**		
WH-NIC N-F	CGTTTGGTGGACCCTCAGAT	WHO Molecular assays to diagnose COVID-19: Summary table of available protocols- National Institute of Health, Thailand
WH-NIC N-R	CCCCACTGCGTTCTCCATT	
WH-NIC N-P	FAM-CAACTGGCAGTAACCA- BHQ1	

Supplementary Table 2: List of synthesised gene fragments used as DNA/RNA template for MCDA, LAMP and rt-PCR. Blue indicates universal M13 adapters while red depicts the sequence for T7 promoter.

Gene fragment name	Position in NC_045512.2	Gene target	Sequence 5' --> 3 '
Region 1	416-931	ORF1ab	TGTAAAACGACGGCCAGTTAATACGACTCACTATAGTGTGGCTTAGTAGAAGTTGAAAAAGGCGTTTTG<br>CCTCAACTTGAACAGCCCTATGTGTTCATCAAACGTTCGGATGCTCGAACTGCACCTCATGGTCATGTTA<br>TGGTTGAGCTGGTAGCAGAACTCGAAGGCATTCAGTACGGTCGTAGTGGTGAGACACTTGGTGTCCTTG<br>TCCCTCATGTGGGCGAAATACCAGTGGCTTACCGCAAGGTTCTTCTTCGTAAGAACGGTAATAAAGGAG<br>CTGGTGGCCATAGTTACGGCGCCGATCTAAAGTCATTTGACTTAGGCGACGAGCTTGGCACTGATCCTT<br>ATGAAGATTTTCAAGAAAACTGGAACACTAAACATAGCAGTGGTGTTACCCGTGAACTCATGCGTGAGC<br>TTAACGGAGGGGCATACACTCGCTATGTCGATAACAACTTCTGTGGCCCTGATGGCTACCCTCTTGAGT<br>GCATTAAAGACCTTCTAGCACGTGCTGGTAAAGCTTCATGCACTTTGTCCGAACAACTGGACTTTATTG<br>GTCATAGCTGTTTCCTG
Region 2	12869-13388	ORF1ab	TGTAAAACGACGGCCAGTTAATACGACTCACTATAGACTGGTACTATCTATACAGAACTGGAACCACCT<br>TGTAGGTTTGTTACAGACACACCTAAAGGTCCTAAAGTGAAGTATTTATACTTTATTAAAGGATTAAAC<br>AACCTAAATAGAGGTATGGTACTTGGTAGTTTAGCTGCCACAGTACGTCTACAAGCTGGTAATGCAACA<br>GAAGTGCCTGCCAATTCAACTGTATTATCTTTCTGTGCTTTTGCTGTAGATGCTGCTAAAGCTTACAAAG<br>ATTATCTAGCTAGTGGGGGACAACCAATCACTAATTGTGTTAAGATGTTGTGTACACACACTGGTACTG<br>GTCAGGCAATAACAGTTACACCGGAAGCCAATATGGATCAAGAATCCTTTGGTGGTGCATCGTGTTGTC<br>TGTACTGCCGTTGCCACATAGATCATCCAAATCCTAAAGGATTTTGTGACTTAAAAGGTAAGTATGTAC<br>AAATACCTACAACTTGTGCTAATGACCCTGTGGGTTTTACACTTAAAAACACAGTCTGTACCGTCTGCG<br>GTAGGTCATAGCTGTTTCCTG
Region 3	28246-28747	N	TGTAAAACGACGGCCAGTTAATACGACTCACTATAGTAGATTTCATCTAAACGAACAAACTAAAATGTC<br>TGATAATGGACCCCAAAATCAGCGAAATGCACCCCGCATTACGTTTGGTGGACCCTCAGATTCAACTGG<br>CAGTAACCAGAATGGAGAACGCAGTGGGGCGCGATCAAAACAACGTCGGCCCCAAGGTTTACCCAATA<br>ATACTGCGTCTTGGTTCACCGCTCTCACTCAACATGGCAAGGAAGACCTTAAATTCCCTCGAGGACAAG<br>GCGTTCCAATTAACACCAATAGCAGTCCAGATGACCAAATTGGCTACTACCGAAGAGCTACCAGACGA<br>ATTCGTGGTGGTGACGGTAAAATGAAAGATCTCAGTCCAAGATGGTATTTCTACTACCTAGGAACTGGG<br>CCAGAAGCTGGACTTCCCTATGGTGCTAACAAAGACGGCATCATATGGGTTGCAACTGAGGGAGCCTT<br>GAATACACCAAAAGATCACATTGGCACCCGCAATCCTGCTAACAATGCTGCAATCGTGGGTCATAGCTG<br>TTTCCTG

BLUE = universal M13
RED = T7 promoter
